# The JCVI standard operating procedure for annotating prokaryotic metagenomic shotgun sequencing data

**DOI:** 10.4056/sigs.651139

**Published:** 2010-03-30

**Authors:** David M. Tanenbaum, Johannes Goll, Sean Murphy, Prateek Kumar, Nikhat Zafar, Mathangi Thiagarajan, Ramana Madupu, Tanja Davidsen, Leonid Kagan, Saul Kravitz, Douglas B. Rusch, Shibu Yooseph

**Affiliations:** 1 J. Craig Venter Institute, Rockville, MD 20850; 2 J. Craig Venter Institute, San Diego, CA 92121

**Keywords:** J. Craig Venter Institute, prokaryotic shotgun metagenomics, environmental sequencing, functional annotation, Global Ocean Sampling, Sargasso Sea

## Abstract

The JCVI metagenomics analysis pipeline provides for the efficient and consistent annotation of shotgun metagenomics sequencing data for sampling communities of prokaryotic organisms. The process can be equally applied to individual sequence reads from traditional Sanger capillary electrophoresis sequences, newer technologies such as 454 pyrosequencing, or sequence assemblies derived from one or more of these data types. It includes the analysis of both coding and non-coding genes, whether full-length or, as is often the case for shotgun metagenomics, fragmentary. The system is designed to provide the best-supported conservative functional annotation based on a combination of trusted homology-based scientific evidence and computational assertions and an annotation value hierarchy established through extensive manual curation. The functional annotation attributes assigned by this system include gene name, gene symbol, GO terms, EC numbers, and JCVI functional role categories.

## Introduction

Shotgun metagenomics sequencing datasets are among the most challenging types of biological information to successfully handle from the perspectives of both size and complexity [1]. Nonetheless, they represent the best method available for sampling the largely uncharacterized diversity of microbes (and their array of functional genes) present in environmental samples [2,3]. In this context, the term “environmental” includes both traditional (e.g., soil, water, air) and host-based (e.g., oral biofilm, distal gut) samples. Moreover, this technique greatly enables the study of uncultivated organisms, which represent the vast majority of life in a number of biomes, including an astonishing 99% in soil [4].

The issue of how to handle this diversity and complexity became especially pressing at the time of JCVI’s Sargasso Sea and Global Ocean Survey expeditions [1,5], which each produced and required the analysis of several million Sanger reads. Although these data sets were revolutionary in their size at the time, the switch from Sanger to next-generation sequencing technologies for the study of environmental complexity has caused metagenomics data sets to become exponentially larger and more complex [6,7]. Using the JCVI prokaryotic metagenomics analysis pipeline, we routinely process data an order of magnitude larger than the original Sargasso collection.

In order to maximize scientific flexibility, the prokaryotic metagenomics pipeline, as reported here, is compartmentalized into structural and functional annotation components, which can be run together or separately. It is designed to process data sets of the scale of tens of millions of sequencing reads given JCVI’s current computing resources, and is scalable to even larger data sets.

There are a number of systems other than the JCVI metagenomics pipeline designed for the purpose of rapid and consistent functional annotation of environmental shotgun sequencing data, including MG-RAST [8] and IMG/M [9].

## Requirements

The JCVI prokaryotic metagenomics pipeline is designed for significant input flexibility. Gene finding, which is referred to in this paper as *structural annotation*, requires as input a multi fasta file containing nucleotide sequence, while the *functional annotation* component accepts multi fasta inputs of peptide sequence. The various structural and functional annotation activities also rely on the presence of sequence, profile, and HMM databases (e.g., Pfam, TIGRFAM) for comparison, as described in the appropriate sections below.

## Procedure

**Figure 1:**
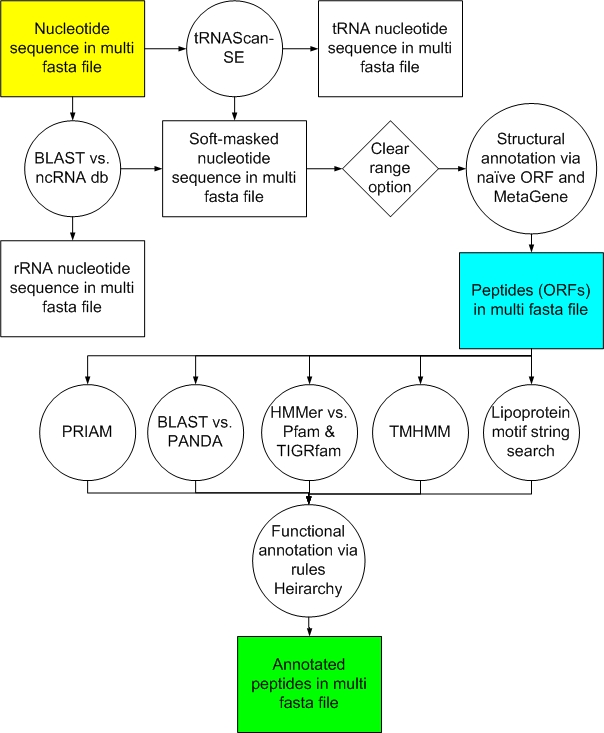
Metagenomics annotation process diagram. This overview covers both the structural (yellow through blue) and functional (blue through green) components of the JCVI prokaryotic metagenomics processing pipeline. Attributes assigned include common name, gene symbol, EC number, GO term, JCVI role category, along with transmembrane character and lipoprotein motifs, as applicable.

The system is compartmentalized into structural and functional annotation components. These can be operated independently or together to meet the scientific objectives. The process as a whole is diagrammed in Figure 1.

Structural annotation results in the identification of the most probable proteins or fragments thereof, present in nucleotide sequence data. It also produces lists of ncRNAs. Functional annotation, the assignment of functional attributes to putative protein sequences, is derived based on a value hierarchy established via homology to the corpus of available resources using BLAST [10], RPS-BLAST [11], HMM [12], and other homology search algorithms. The primary attributes assigned by the functional annotation component are: gene name, gene symbol, GO terms [13], EC numbers [14], and JCVI functional role categories [15]. The EC system is a numerical classification scheme based on the chemical reaction(s) that a specific protein catalyzes [14], while GO terms seek to impose structured controlled vocabularies to describe molecular functions, biological processes, and cellular components [13]. JCVI role categories are a two-level functional classification system of assignments for protein cellular function [15].

The final output of this pipeline can be visualized in either tab delimited flat-file annotation summary format or using in-house visualization tools (unpublished). Intermediate results, including those from BLAST and HMM analyses are also persisted and can be used to dig deeper into the data. A complete list of third party programs utilized in this system, as well as invocation parameters thereof, can be found in Table 1.

**Table 1:** Third party tools, cutoffs, and parameters used in this pipeline

**Process**	**Function**	**Tool**	**Parameters**
Structural Annotation			
	tRNA identification	tRNAscan-SE (1.23)	tRNAscan-SE -q -b -G
	ncRNA finder stage 1	BLAST	blastall -p blastn -i -d -e 0.1 -F "T" -b 1 -v 1 -z 3000000000 -W 9
	ncRNA finder stage 2	BLAST	blastall -p blastn -i -d -e 1e-4 -F "m L" -b 1500 -v 1500 -q -5 -r 4 -X 1500 -z 3000000000 -W 9 -U T
	Protein Identification	MetaGeneAnnotator	-m
Functional Annotation			
	Protein annotation	BLAST	blastall -p blastp -v 10 -b 10 -X 15 -e 1e-5 -M BLOSUM62 – J F -K 10 -f 11 -Z 25.0 -W 3 -U F -I F -E -1 -y 7.0 -G -1 –A 40 -Y 0.0 -F "T" -g T -d -i -o -z 1702432768 -m 7
	Protein annotation	hmmpfam	
	Protein annotation	lipoprotein_motif	--is_micoplasm 0
	Protein annotation	tmhmm	
	EC assignment	PRIAM	rpsblast -i -d -m 8 -e 1e-10

## Structural Annotation

The function of this component is to identify the best possible open reading frames from the metagenomics shotgun sequencing reads. This is performed in full knowledge that the putative proteins identified are likely to be fragments of the full-length protein – and as such the beginning and end of each read are treated as putative start and stop sites. This process can be run with the “clear-range” mode either on or off; the former mode is useful primarily for Sanger data. In this case, only the region of each trace specified by the clear-range information in the fasta header is used in the analysis. Clear-ranges are established using the base correctness metric “quality values” [16,17].

Prior to identification of protein coding genes in the sequence data, ncRNAs must first be found and masked. This is accomplished through a pair of processes, tRNAScan-SE [18] and a set of two increasingly stringent BLAST [10] searches performed against a JCVI’s internal reduced-complexity rRNA database (Table 1). The latter contains a representative sampling of known 5S, 16S, 18S, and 23S rRNA sequences. In both cases, the identified regions are “soft-masked”, and the ncRNAs written to separate output multi fasta files for downstream rRNA analyses. Soft-masking is a method used by the structural annotation pipeline to prevent information loss at the read level while allowing certain zones of the fasta record to be differentiated. Converting the region of the sequence containing ncRNAs to lower case letters transfers enough information downstream to exclude ncRNAs before putative proteins are identified.

The resulting soft-masked sequences are subjected to a three step putative protein identification process. Step 1 is a naïve 6-frame translation that identifies each possible ORF with a minimum size of 180 nucleotides. This cutoff was selected based on the expected size of typical bacterial genes (~900 bp, unpublished), such that a reasonable fraction (~20%) of a putative gene is the minimum for annotation to proceed. Note that smaller cutoffs can be used as needed, such as in the case of viral metagenomics processing. Each run of the pipeline requires a user specified codon table that determines the length and actual amino acid sequence of each ORF. ORFs are defined as the longest possible frame from start to stop. The beginning and end of each sequence record is treated as both stop and start, for purposes of maximizing the sensitivity of the system for fragments. Step 2 requires the use of MetaGeneAnnotator [19], an *ab initio* gene predictor tool which uses empirical data including sequence base composition, distance, and orientation of genes of completely sequenced genomes to identify open reading frames. Step 3 consists of using the putative proteins identified in step 2 to “tag” the ORFs found in step 1 – those overlapping the nucleotide space of the MetaGene calls are defined as the most likely proteins, even if they extend past the defined MetaGene prediction boundary. This process produces set of the longest possible putative proteins from sequence data. The output produced is a multi fasta file of putative peptides for functional annotation. Overlaps between ncRNAs and putative proteins are allowed if they compose less than 30 of the 180 nucleotide minimum (i.e., 150 unmasked nucleotides required for structural annotation). The results of the JCVI structural annotation pipeline are frequently supplemented by putative proteins identified through incremental clustering processing of the same sequence data [20]. The putative proteins are processed by the functional annotation component.

Unlike MG-RAST, our pipeline does not confine itself to BLASTX based peptide identification using a defined dataset, and as a result has a larger yield of putative proteins for an equivalent number of input reads. IMG/M, on the other hand, takes a two-tiered approach, with the proteins in reads assessed directly using RPS-BLAST against Pfam and COG [21] databases, and indirectly through BLASTX-derived “proxygenes” [22]. Both IMG/M and MG-RAST thus consolidate the structural and functional searches into a single step, while the JCVI system obtains additional flexibility by separating them.

## Functional Annotation

Functional annotation, the assignment of the most probable biological role for a given peptide, occurs in two phases: the collection of a wide array of information for each putative protein (“data collection components”), and the application of an annotation value hierarchy to that information corpus. In such a way, each putative protein is given annotation that can be conservatively supported by the available collection of homology-based scientific knowledge. For all the steps below, the pipeline provides raw outputs that may be used for downstream analysis. Note that the data collection components operate independently and in parallel, but the value hierarchy operation must wait on completion of all the data collection components to proceed to functional assignment.

The collection of functional attributes on each putative protein begins with the use of BLASTP against the most recent version of JCVI’s PANDA data resource, an internal collection of non-redundant protein and nucleotide data sets derived from a variety of public databases (e.g., NCBI GenBank, UniProt) on an ongoing basis. This analysis includes the use of a BLOSUM62 substitution matrix [23] and an E value cutoff of 1e-5 (Table 1). For each peptide, the 10 most significant alignments are stored. The classes of BLAST hits defined in this process are delineated and ordered in Table 2. This step produces two output format types, one is a tab delimited format with each alignment represented by one row, and the other is an XML file. These BLAST XML files can be imported into the MEGAN [24], or other comparable tools, for phylogenetic analysis if desired.

**Table 2:** BLASTP evidence classes.

**BLAST Hit Class**	**Annotative meaning**
High Confidence	35% identity or greater, across 85% or more of the length
Putative	less than 35% identity, but across 80% or more of the length
Conserved Domain	35% identity or greater, but across less than 80% of the length
Low Confidence	less than 35% identity across less than 80% of the length

These have been established through extensive manual curation and validation efforts, and are ordered by trustworthiness.

The second data collection component is the search against global (ls) HMM models in the current releases of Pfam [25] and TIGRFAM [26]. This search is conducted using the SIMD-accelerated HMMer2-like functionality provided by CLC Biosciences Computational Cell [27]. In all cases, standard trusted cutoffs are used. The HMM hits are organized into ordered isology type classes (“isotypes”), as listed in Table 3, each of which represents a different degree of confidence about the functional assignment. In the near term, we will use also incorporate searches against fragment HMM models in the annotation assignment process.

**Table 3:** HMM hit isotype classes.

**HMM Hit Class**	**Annotative meaning**
Equivalog	All proteins scoring above the trusted cutoff have the exact same function
Equivalog Domain	All domains scoring above the trusted cutoff have the same function; can be part of a multi-function protein
Domain	Defines a region of homology that may or may not have a known function, and need not be the full length of the polypeptide
Subfamily	Hits in this category represent full-length homology within a subgroup comtained within a gene family
Superfamily	This defines a set of proteins with full-length homology of sequence and domain architecture, but not necessarily the same function
Hypothetical - isotype	Unknown function
Uncharacterized	PFAM model cannot be assigned

JCVI classifies HMMs into more than a dozen categories (isology types, or “isotypes”), each of which represents a different degree of confidence about the functional classification.

The third data collection component involves a RPS-BLAST [11] against the PRIAM database [28] of metabolic enzymes. This is primarily for the purpose of assigning EC#s in those cases without a TIGRFAM hit, which would otherwise take precedence. The cutoff used is 1e-10 (Table 1).

The final data collection component involves searches for lipoprotein motifs and transmembrane helices in the putative proteins. The former is accomplished using a regular expression search in the amino acid sequence, while the latter is performed using TMHMM [29], a HMM-based search for transmembrane motifs (Table 1). These two searches represent annotation states that fall well short of complete functional annotation (e.g., “putative lipoprotein”), but are more informative than the absence of any functional annotation.

Annotation is assigned using the hierarchical scheme detailed in Table 4, which was derived from JCVI’s extensive experience in the manual curation of prokaryotic genomes. Note that in each case, there is a balance struck between the most trusted annotation and that which provides the most scientific value. The hierarchy applies primarily to common name and gene symbol attributes, with EC numbers, GO terms, and JCVI role categories handled in a more nuanced way. Gene Symbols are assigned based on top blast hits to a curated internal prokaryotic database called OMNIOME [30]. EC numbers and JCVI roles are assigned via TIGRFAM hits in preference to Pfam hits. EC numbers are also assigned by PRIAM searches but we do not assign common name or symbol based on this evidence type. In the case of GO terms, these are assigned in the following evidence order: TIGRFAM, EC number, Pfam, and PANDA. In the case of PANDA, the top ten blast hits are scored based on any hit to OMNIOME [30] which is then used to assign GO terms.

**Table 4:** Metagenomics annotation hierarchy.

**Annotation Rank**	**Evidence Type**	**Evidence Class**
1	HMM	TIGRfam Equivalog
2	HMM	Pfam Equivalog
3	HMM	TIGRfam Hypothetical Equivalog
4	HMM	Pfam Hypothetical Equivalog
5	HMM	TIGRfam Domain
6	PRIAM	PRIAM
7	HMM	TIGRfam Subfamily
8	HMM	TIGRfam Superfamily
9	HMM	TIGRfam EquivalogDomain
10	HMM	TIGRfam Hypothetical Equivalog Domain
11	HMM	TIGRfam Subfamily Domain
12	HMM	Pfam Subfamily
13	HMM	Pfam Superfamily
14	HMM	Pfam Equivalog Domain
15	HMM	Pfam Hypothetical Equivalog Domain
16	HMM	Pfam Subfamily Domain
17	BLAST	Panda BLASTP High Confidence
18	HMM	TIGRfam Domain
19	HMM	Pfam Domain
20	HMM	Pfam Uncharacterized
21	BLAST	Panda BLASTP Putative
22	BLAST	Panda BLASTP Conserved Domain
23	TMHMM	TMHMM
24	LIPOPROTEIN	Lipoprotein Motif
25	DEFAULT	Hypothetical

This hand-curated and validated list, derived from years of experience with prokaryotic genome analysis, allows for the best-supported conservative functional annotation based on the available homology-based evidence and computational assertions.

Putative proteins without any evidence but identified by MetaGeneAnnotator [19] are classified as “hypothetical”. This set of non-conserved hypothetical peptides usually constitutes at least 30% of all putative proteins in a dataset.

The header format for the multi fasta output from the functional annotation pipeline can be seen in Table 5.

**Table 5:** Output format of the JCVI prokaryotic metagenomics functional annotation multi fasta file header, with example entries.

**Column Header**	**Example Entry**
User Id	GCA1659448.b1
Peptide Id	JCVI PEP 5160785.1
Common Name Section Starts	common name
Common Name	glutamine synthetase, catalytic domain
Common Name Evidence	PF00120
Gene Symbol Section Starts	gene symbol
Gene Symbol	glnT
Gene Symbol Evidence	RF|YP 266724.1|71084004|NC 007205
GO Section Starts	GO
GO Terms	GO:0004356 // GO:0006542
GO Term Evidence	PF00120 // PF00120
EC Section Starts	EC
EC Id	6.3.1.2
EC evidence	PF00120
TIGR Role Section starts	TIGR role
Tigr Role Id	73
Tigr Role Evidence	PF00120

## Implementation

The prokaryotic metagenomics pipeline is divided into structural and functional annotation components, which can be run together or separately. The underlying codebase itself is divided in two distinct sections. The first section is written in Java, and leverages JCVI's high-throughput computing platform, which provides a framework for scalable and robust implementations of data analysis pipelines. This software platform itself is built on top of JBoss - an open-source JEE server designed to allow for easy failover setup and clustering. Layered design, strict adherence to standard interfaces such as JMS, EJB, Web Services, and high level of adoption of standard open source packages (e.g., Hibernate, DRMAA) ensures platform stability and ease of integration with other software packages. Other built-in capabilities include a robust workflow subsystem, grid integration tier, and a growing set of bioinformatics tools implemented to scale to modern data and compute requirements. The remaining portion of this application lies within the PERL codebase, which includes parsers of all the raw results and the execution of the hierarchical annotation algorithm, all of which are invoked from within the Java portion.

## Discussion

The progress of metagenomics as a field requires both that consistent, high quality functional annotation be achievable in a timely manner, and that new computational methods to improve those functional assignments be incorporated. The JCVI prokaryotic metagenomics analysis pipeline provides an efficient system for identifying and functionally classifying the proteins present in shotgun metagenomics sequencing data for sampling communities of prokaryotic organisms. This prokaryotic pipeline complements ongoing activity in viral metagenomics annotation also underway at JCVI.

It is JCVI’s intention to continually upgrade this tool to take advantage of the not only the newest versions of existing resources, but to incorporate new resources and technologies (e.g., cloud computing) as they become available. It is relevant to note that reproducibility of the results produced by this system depends substantially on the versions of the databases underlying the pipeline (e.g., PANDA, Pfam). As these data resources are iteratively updated over time with newer versions, both a net improvement in functional assignments and cumulative decrease in comparability between older and newer data sets are expected.

It is the intention of JCVI to make this resource available to the scientific community in the near future.
